# Shift rotation, circadian misalignment and excessive body weight influence psychomotor performance: a prospective and observational study under real life conditions

**DOI:** 10.1038/s41598-019-55114-w

**Published:** 2019-12-18

**Authors:** Dayane Eusenia Rosa, Luisa Pereira Marot, Marco Túlio de Mello, Fernanda Veruska Narciso, Bruno da Silva Brandão Gonçalves, Elaine Cristina Marqueze, Cibele Aparecida Crispim

**Affiliations:** 1 Faculty of Medicine, Federal University of Uberlandia, Uberlândia, postcode: 38405-320 Brazil; 2 Department of Sports, Faculty of Physical Education, Physiotherapy and Occupational Therapy, Federal University of Minas Gerais, Belo Horizonte, postcode: 31310250 Brazil; 3 School of Arts, Sciences and Humanities, University of São Paulo, São Paulo, postcode: 03828-000 Brazil; 4 Catholic University of Santos, Santos, postcode: 11015002 Brazil

**Keywords:** Circadian regulation, Neurophysiology, Health occupations

## Abstract

We aimed to evaluate the influence of shift work rotation, circadian misalignment and being overweight/obese on psychomotor performance throughout a complete shift rotation schedule. The study was conducted with 30 males working rotating shifts from a mining company under real life conditions. Individuals were evaluated over seven days in a shift schedule carried out as follow: two shifts in the morning (D1 and D2), two shifts in the afternoon (D3 and D4), 24 hour free day (D5) and two shifts at night (D6 and D7). Work performance was evaluated by psychomotor vigilance task tests (PVT), and actigraphy was used to characterise the rest-activity rhythm based on intradaily variability (IV) and interdaily stability (IS) of nonparametric functions. We found a significant effect of the shift, body mass index (BMI), IS and IV on lapses in attention. More lapses occurred on D7 than D1, D2, D3 and D4 of the schedule shift. The obese group presented a higher number of lapses in attention than eutrophic. The interaction between day and IS showed that less synchronised individuals presented a higher number of lapses in attention on D7 than D1 and, for the interaction between day and IV, more fragmented individuals presented a higher number of lapses in attention on D7 than D6. We conclude that higher BMI, lower synchronisation and higher fragmentation of the rest-activity pattern influenced lapses in attention throughout the shift rotation.

## Introduction

Shift work is characterised by 24 hours of operations and presents a wide variety of working time arrangements, including all working hours that are outside the normal daytime ones^1^. In industrialised society it is estimated that this type of work represents more than 20% of employees^2^, in the USA it is 28.7%^3^ and in Brazil the percentage for shift work has not yet been calculated, but specific data for night work shows a percentage of 15%^4^. Among the different types of shift work arrangements, the rotating shift – characterised by constant alternation of working hours and free days within a pre-established period^5^ – is quite common among companies^6^. The direction of shift schedule rotation (clockwise or counterclockwise) and rotation speed (fast or slow) also presents variation among shift work schedules^7^.

The physiology and behaviour of human beings are coordinated by an intrinsic molecular clock into rhythms that are synchronised with the 24 hour solar day. The suprachiasmatic nucleus (SCN), the primary synchroniser, works with various peripheral synchronisers^8^, and both are influenced by external time cues – such as light-dark, meals and social interaction^9^. Shift work, mainly night shifts, is impacted by most of these external synchronisers – such as the daytime pattern of exposure to light and the exposure time to synchronisers^10^. Thereby, adjustments in the phase of endogenous circadian rhythms are compromised in shift workers^9^, which leads to desynchronisation between the circadian clock and the sleep cycle, known as circadian misalignment^11^. Currently it is possible to quantify circadian misalignment by assessing variables such as interdaily stability (IS, i.e. the stability of the rhythm over days) and intradaily variability (IV, i.e. the fragmentation of the rhythm relative to its 24 hour amplitude) by actigraphy^12^.

Circadian misalignment may negatively affect psychomotor performance, since cognitive functions such as vigilance level and, consequently, the development of lapses in attention are directly linked to circadian and homeostatic influences, which in turn suffer fluctuations throughout the day^13^. The physiological mechanism involving wakefulness levels is determined by responses generated by the parasympathetic and sympathetic nervous systems through environmental stimuli^14^. Therefore, the vigilance level of individuals fluctuates over the course of a day^14^ and may be impacted by misalignment. This can lead to an inappropriate response, indicating a state of decompensation and failure of physiological functions^15^. It has been documented that approximately 10% to 30% of fatal traffic deaths are due to sleepiness and fatigue, which are directly associated with lapses in attention^16^. In addition, variables such as the workload and the nutritional status of individuals have been shown to be possible variables that can alter psychomotor performance^17,18^.

Previous studies showed that circadian misalignment also leads to losses in physiologic functions, which show a 24 hour rhythmicity and are ruled by the light/dark cycle, such as the sleep/awake cycle^19^ and macronutrients metabolism^20,21^. In this condition, the release of hormones associated with food consumption, such as leptin and ghrelin, may be altered, which establishes a link between circadian misalignment and obesity^22^. Cook *et al*.^23^ postulated that even in situations where there is no apparent circadian misalignment, obesity is capable of interfering with psychomotor performance^23^. However, the relationship between psychomotor performance, circadian misalignment and obesity is little explored among shift workers.

Rotating shift workers experience a large variation in working times over a short period. In this sense, it is reasonable to assume that circadian misalignment leads to diminished work performance, and in obese subjects this situation could be worse^24^. This study aimed to evaluate the influence of a rotating shift, circadian misalignment and being overweight/obese on the performance of rotative shift workers. We hypothesised that excessive body weight, lower synchronisation and higher fragmentation of the rest and activity pattern can lead to an increase in the number of lapses in attention throughout the scheduled shift days in rotative shift workers.

## Methods

### Participants and ethics

This prospective, observational study was conducted with 30 males that worked for a mining company located in a city in the midwest of Brazil, on a rotative shift work schedule. The shift workers were informed about the objectives and procedures, and after that they were invited to join the study. This study was approved by the Human Research Ethics Committee of the Federal University of Uberlândia (CAAE: 49689115.0.0000.5152), all methods were performed in accordance with the relevant guidelines and regulations and all subjects signed an informed consent form.

Participants were selected if they met the following criteria: works a clockwise rotating shift (morning-afternoon-night); works in an operations control panel or leadership position; is able to wear actigraphy monitors and to perform the psychomotor performance tests (PVT); has not done trans-meridian travel in the three months prior to the start of the study. Participants were excluded if they had a sleep disorder or other comorbities (heart and metabolic diseases, cancer, neurological and degenerative brain diseases), hearing and visual impairments, had loss of consciousness or used medication capable of inducing sleep or wakefulness. This information was obtained from the occupational medicine area of the company where the study was conducted and by the initial questionnaire and sleep diary. From the company’s total workforce, 30 subjects (aged between 25 and 52 years old) met the inclusion and exclusion criteria and agreed to participate in the study.

On the first day of the study, initial evaluations such as questionnaires regarding sociodemographic and health behaviours, as well as anthropometric and biochemical analyses, were conducted. Then, individuals were evaluated over seven days regarding their work performance (PVT) and rhythm rest-activity pattern (actigraphy).

### Initial Evaluation

#### Sociodemographic data and health behaviours

All subjects answered a questionnaire about sociodemographic aspects, such as age, marital status, presence of children, level of education and years of shift work, as well as health behaviours such as frequency of physical activity, alcohol intake, smoking habits, diseases diagnosed and use of medicines.

#### Blood parameters

The subjects were instructed to fast overnight for 12 hours before blood sample collection. All procedures occurred at the company’s ambulatory clinic. The biochemical analyses measured were: fasting blood glucose, fasting insulin, insulin resistance index (HOMA-IR), total cholesterol, low density lipoprotein (LDL-c), high density lipoprotein (HDL-c) and triglycerides (TG).

The glucose-oxidase method (Siemens, Chicago, IL, USA) was used to determine the glucose serum concentrations. Insulin concentrations were accessed using a commercial enzyme-linked immunosorbent assay kit (ELISA) (Siemens). Homeostasis model for the assessment of insulin resistance (HOMA-IR) was determined using the following formula described by Matthews *et al*.^25^, fasting serum insulin (μIU/L) × fasting serum glucose (mmol/L)/22.5. The concentrations of cholesterol, triglycerides and HDL-cholesterol were determined by means of coupled reactions. The products of this reaction are a coloured complex that can be measured by spectrophotometry. Each of the specific analyses was accessed using a commercial kit from Biosystems® (cholesterol - Ref. 21505, triglycerides - Ref. 11828, HDL-cholesterol - Ref. 11648). The value of LDL-cholesterol was determined using the formula described by Friedewald^26^.

#### Anthropometric variables

Weight and height measurements were performed according to the standardisation method proposed by Lohman *et al*.^27^ Weight was measured with a high precision scale accurate to 0.1 kg (Toledo Scale Corp., Toledo, Ohio). To measure height, a stadiometer coupled to a scale with an accuracy of 0.1 cm (Toledo Scale Corp., Toledo, Ohio) was used. Waist circumference (WC) was measured as the midpoint between the last rib and the iliac crest using an inelastic measuring tape^28^. According to World Health Organization recommendations, a WC ≥ 102 cm was considered abdominal obesity^29^. Body mass index (BMI, kg/m²) was calculated as the weight (kg) divided by the height squared (m^2^). A BMI <25 kg/m² was considered eutrophic, ≥25 to <30 kg/m² overweight and ≥30 kg/m² obese^29^.

#### Evaluations conducted over shift schedule

**Figure 1:**
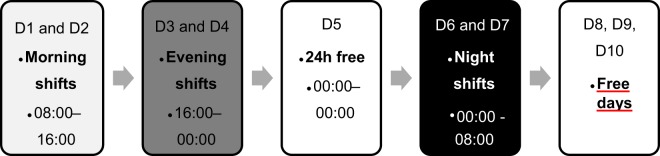
Shift schedule days. D = shift days; working times of each shift = morning (D1 and D2) – 08:00 to 16:00; afternoon (D3 and D4) – 16:00 to 00:00; 24 h free day (D5) – 00:00 to 00:00; and night (D6 and D7) – 00:00 to 08:00.

Individuals were followed for seven consecutive days carried out as follows: two days (D1 and D2) working during the morning (08:00–16:00); two days (D3 and D4) working during the evening (16:00–00:00); two days (D6 and D7) working during the night (00:00–08:00). On the 24 hours of rest (D5) between the last day of evening shift and the first one at night, as well as on free days (D8, D9 and D10), the psychomotor vigilance test (PVT) was not performed (Fig. 1).

#### Actigraphy

The actigraphy data were collected using the actigraph - Act trust (Condor Instruments®). This tool was configured for collecting information every 24 hours over the period of 15 consecutive days. The information was downloaded through the software ActStudio (Condor Instruments® - version 1.0.0.0050.2015). The volunteers wore the actigraph on their non-dominant wrist. Use of the actigraph was recommended during two complete shift schedules and volunteers filled out a sleep diary with information on their activity during this period^30^. For this paper we analysed only data from the period between D1–D7 (on the first shift rotation). The correct use of actigraphy were certificated by cellphone messages. The actigraph is a tool validated to identify the patterns of circadian rhythm during shift work^31^. Through the analysis of circadian rhythm variables by the cosinor method it is possible to obtain information about parametrical (acrophase, mesor, period and amplitude) and nonparametric variables for quantifying the stability and fragmentation of the rest-activity rhythm^32^.

In this research, rhythm fragmentation was measured by intradaily variability (IV), while synchronisation to the 24 hours light-dark cycle was measured by interdaily stability (IS)^33^. The IV provides information on rest activity rhythm fragmentation. The IV calculation is based on the first derivate of the hourly clustered actimetry data (Eq. (1)). The first derivate is the result of subtracting the previous element (Xi_1) from the posterior element (Xi) of the raw data. From the first derivate, the root mean square is calculated and the result normalized by the raw data population variance. Large hourly differences such as daytime sleep or nighttime awakenings increase the value of IV. High IV values indicate a fragmented rhythm, such as the presence of daytime sleep and/or nocturnal awakenings (Witting *et al*., 1990).

1 $${\rm{IV}}=\,\frac{{\sum }_{{\rm{i}}=2}^{{\rm{N}}}{({{\rm{X}}}_{{\rm{i}}}-{{\rm{X}}}_{{\rm{i}}-1})}^{2}{\rm{N}}}{({\rm{N}}-1){\sum }_{{\rm{i}}=1}^{{\rm{N}}}{({{\rm{X}}}_{{\rm{m}}}-{{\rm{X}}}_{{\rm{i}}})}^{2}}$$

Interdaily stability (IS), which yields information about the rest activity rhythm synchronization with the light dark cycle, is calculated from the mean 24-h profile (24 points represent the hours of the day). In Eq. 2, N corresponds to the total number of data items, p is the number of data items per day (24 in this case), Xm is the average of all data, Xh corresponds to each hour of the mean profile, while Xi represents each given hour of raw data. IS is calculated as the variance of the average daily profile divided by the total variance.

2 $${\rm{IS}}=\,\frac{{\sum }_{{\rm{h}}=1}^{{\rm{p}}}{({{\rm{X}}}_{{\rm{h}}}-{{\rm{X}}}_{{\rm{m}}})}^{2}{\rm{N}}}{({\rm{p}}){\sum }_{{\rm{i}}=1}^{{\rm{N}}}{({{\rm{X}}}_{{\rm{i}}}-{{\rm{X}}}_{{\rm{m}}})}^{2}}$$

We used the median values of IS (0.23) and IV (0.66) to create two groups: for IS, less synchronised (≤0.23) or more synchronised (>0.23); and for IV, less fragmented (≤0.66) or more fragmented (>0.66). Fragmentation (IV) may be derived from daytime sleepiness and/or nocturnal arousals. Higher IV values are related to worse sleep efficiency. The variable IS indicated it was synchronised with the 24 hour zeitgeber. High values show that the subject was synchronised. This reflects good functioning of the CTS components related to photic and nonphotic synchronisation (social effects and schedule shifts^33^).

#### Psychomotor vigilance performance

Psychomotor vigilance performance was measured using a portable psychomotor vigilance task (PVT) model 192 (Ambulatory Monitoring, Inc., NY). The PVT tests had durations of five minutes and occurred just before and after the work period, over six days of shift work (corresponding to a complete work schedule) at the workplace. The participant performed the test alone in a quiet room (before the PVT test all partipants were trained to use the machine). The PVT-5 min was used to measure sustained attention and psychomotor vigilance of participants. The variable analysed for this study was mean number of lapses of attention. Lapses of attention are disruptions in performance that typically last reaction time (RT) > 500 ms^34^. The protocol adopted only used visual response tests, during which bright red visual stimuli (from a light-emitting diode [LED] digital counter) were flashed at intervals of 2 to 10 seconds on the screen of the device. The participants were instructed to press a response button, located on the right side of the device, as soon as the visual stimuli appeared^35^. The PVT test scores (pre and post work) were summed up and we got the number of lapses of attention (number of lapses of attention = number of lapses of attention pre work + number of lapses of attention post work). The values obtained were analysed using the software Microsoft Excel®.

### Statistical analysis

The Shapiro-Wilk test was performed to test the normality of the data. Data are presented as mean and standard error or median and interquartile range. The Spearman correlation test was used to analyse the correlation between lapses in attention and BMI and between IS and IV. The linear regression test was used for continuous variables such as IS, IV and BMI.

Generalised estimating equations (GEE) were used to analyse the single and interacting effects of independent variables (day of the shift schedule, BMI, IS and IV) on the dependent variable (number of lapses of attention). In the present study, an exchangeable correlation structure was used in two models for analysis of the interactions. Model 1 - dependent variable: lapses of attention; independent variables: day of shift work, BMI and IS. Model 2 - dependent variable: lapses of attention; independent variables: day of shift work, BMI and IV. The isolated effects of the independent variables and the interactions between them were tested in both models. Both models were adjusted for age, period of shift work and presence of children at home. Gamma distribution and Sidak sequential test were performed for pairwise comparisons.

Data was assessed for outliers by a visual assessment via scatter plot and SDs from the mean. Three participants with extreme values of lapses were considered as outliers (Outlier 1, Σ number of lapses of attention = 109.0; Outlier 2, Σ number of lapses of attention = 85.1; Outlier 3, Σ number of lapses of attention = 38).

We performed a paired t-test to compare the values between pre- and post-work PVT scores. As we did not find significant differences (p = 0.56), the two values were summed, and total number of lapses of attention of each shift day was considered for the comparisons throughout the complete shift schedule.

In the analysis involving the variables IS and IV, the n sample was 25. The reason for missing data regarding IS and IV variables (n = 5) is because some volunteers did not use the actigraph during the established period and in the correct way as described in the experimental protocol.

Statistical analyses were performed using SPSS version 23.0 (SPSS Inc., Chicago, IL). For statistical significance, α error was set at 5%.

## Results

### Population

Data regarding sociodemographics, clinic, occupational, anthropometric, blood parameters, sleep duration, circadian and psychomotor characteristics are presented in Table 1. Among the thirty individuals evaluated, the majority were married (90%), had children at home (63.3%) and had worked in shifts for 10 years or more (66.7%). Ninety percent of participants did not smoke, while fifty percent drank alcoholic beverages at least once a week and most of the subjects did physical activities at least once a week (63.3%). In regards to anthropometric parameters, the majority of subjects had BMI ≥ 25 kg/m^2^ (76.7%), and 30.0% had abdominal obesity (WC ≥ 102 cm). The individuals had a shorter sleep duration on D5 and during the night shifts (D6 and D7), when compared with morning shifts (D1 and D2), afternoon shifts (D3 and D4) and free days (D8, D9 and D10) (Table 1).

**Table 1:** Sociodemographic characteristics, anthropometric indices, health behaviours, biochemical parameters, sleep duration, variables of rest-activity circadian rhythm and psychomotor performance (PVT) of employees (*n* = 30).

Variables	All (n = 30)
Age (years), mean ± SD	37.2 ± 5.7
Marital status - married, *n* (%)	27 (90.0)
Children at home - < 12 years, *n* (%)	19 (63.3)
***Period of shift work***	
<10 years, n (%)	10 (33.3)
≥10 years, n (%)	20 (66.7)
***Health behaviours***	
Smoking status - Yes, *n* (%)	4 (13.3)
Alcohol intake (at least once a week) - Yes, *n* (%)	15 (50.0)
Physical activity - Yes, *n* (%)	19 (63.3)
***Anthropometric measurements***	
BMI (kg/m^2^), mean ± SD	28.43 ± 3.73
Overweight (BMI ≥ 25 to <30 kg/m^2^), *n* (%)	16 (53.3)
Obese (BMI ≥ 30 kg/m^2^), *n* (%)	9 (30.0)
Waist circumference (cm), median [interquartile range]	96.5 [93.1–100.0]
Abdominal obesity (≥102 cm) - Yes, *n* (%)	9 (30.0)
***Biochemical exams***	
Glucose (mg/dL), mean ± SD	92.1 ± 13.5
Insulin (UI/mL), median [interquartile range]	6.5 [5.1–8.3]
HOMA-IR, median [interquartile range]	1.6 [1.1–1.78]
HDL (mg/dL), median [interquartile range]	38.4 [32.4–43.6]
LDL (mg/dL), median [interquartile range]	98.5 [69.4–117.0]
Triglycerides (mg/dL), median [interquartile range]	113.0 [96.9–140.7]
Total cholesterol (mg/dL), mean ± SD	171.4 ± 37.6
***Sleep duration***	
Morning shifts - D1 and D2 (hour), mean ± SD	7:34 ± 0:13^a^
Afternoon shifts - D3 and D4 (hour), mean ± SD	6:52 ± 0:11^b^
24 hour free day - D5 (h), mean ± SD	4:42 ± 0:20^c^
Night shift - D6 and D7 (h), mean ± SD	5:58 ± 0:11^d^
Free days - D8, D9 and D10 (h), mean ± SD	7:20 ± 0:13^a,b^
Circadian*	
*Interdaily stabilitty, median [interquartile range]*	0.23[0.20–0.27]
*Less synchronized,n (%)*	13 (52.0)
*More synchronized,n (%)*	12 (48.0)
*Intradaily variabilitty, median [interquartile range]*	0.66 [0.58–0.74]
*Less fragmented,n (%)*	13 (52.0)
*More fragmented,n (%)*	12 (48.0)
**Psychomotor vigilance task (PVT)**	
*Number of lapses of attention, median [interquartile range]*	1.00 [1.00–2.00]

Data are expressed as mean ± standard deviation (SD), median [interquartile range] or number (percentage). BMI, body mass index. The sleep duration values with different superscripts are significantly different; *P* < 0.05, calculated by GEE test. *n = 25.

#### Correlation between total number of lapses and BMI

**Figure 2:**
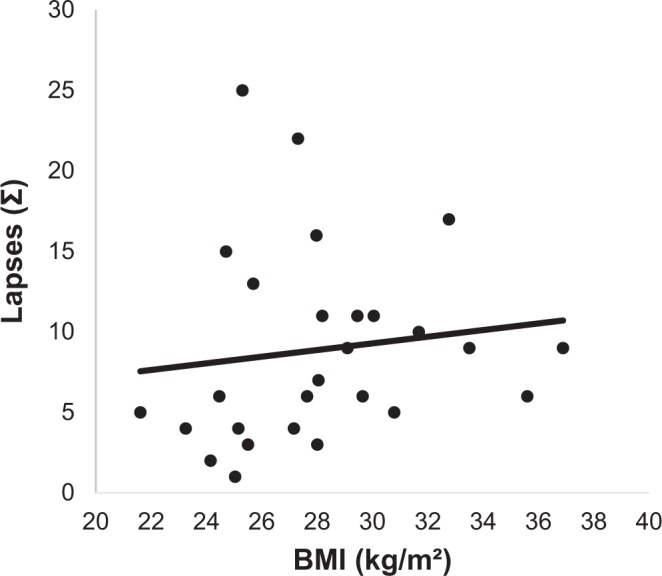
Correlation between total number of lapses in attention in schedule shift and BMI (n = 27; 3 outliers were excluded from this analysis).

We found a significant positive correlation between the total number of lapses in attention during the complete shift schedule (seven days) and BMI (Fig. 2; r = 0.331, *P* < 0.001).

The variables IS and IV usually did not correlate mathematically, which also occurred with the IS and IV data for this study. We performed the correlation test and found no significant results (r = 0.75, p = 0.36).

We compared the PVT scores before and after the work shift and found no significant results to difference between pre- and post- work (p = 0.56).

#### Effect of BMI on the number of lapses of attention

**Figure 3:**
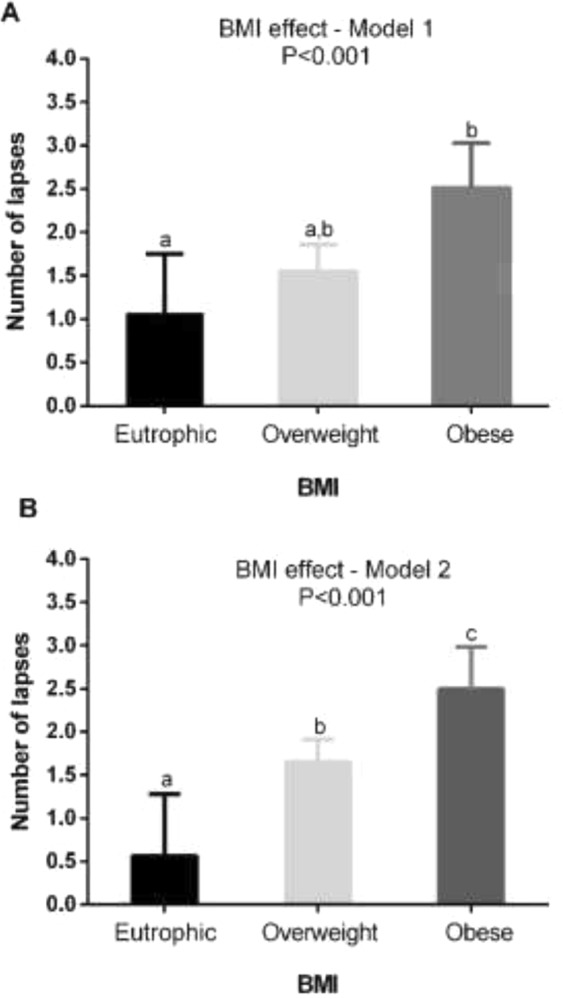
Effect of BMI on number of lapses in attention. (**A**) Model 1 - Effect of BMI on number of lapses in attention through the analysis of GEE and (**B**) Model 2 - Effect of BMI on number of lapses through the analysis of GEE. Data correspond to mean ± standard error of the mean (*n* = 30). The bars with different letters are significantly different; *P* < 0.001. All analysis were adjusted for age, period of shift work and presence of children in the house (see results for statistics).

GEE analysis showed the effect of BMI on the number of lapses in attention. The analysis was conducted through two statistical models: 1) including as independent variables: day of shift, BMI and IS; and 2) including as independent variables: day of shift, BMI and IV. In Fig. 3A (Model 1, including the IS variable), the obese individuals presented a greater number of lapses in attention than eutrophic ones (2.51 ± 0.52 and 1.07 ± 0.68, respectively; *P* < 0.001). In Fig. 3B (Model 2, including IV variable), eutrophic individuals (0.56 ± 0.72) also had a lower number of lapses in attention when compared to overweight and obese individuals (1.65 ± 0.26 and 2.49 ± 0.49, respectively; *P* < 0.001 for all) (Fig. 3B).

The results of Model 1 showed isolated effects of the variables as well as their interactions. In relation to the day of the shift rotation, the highest value of lapses in attention was found on D7 (2.68 ± 0.35; night shift), which was significantly higher than D1, D2, D3 and D4 (0.56 ± 0.77, 1.84 ± 0.34, 1.99 ± 0.29 and 1.69 ± 0.28, respectively; *P* < 0.05 for all). For the effect of synchronisation of the rest-activity rhythm (IS) on the number of lapses in attention, we found that poorly synchronised subjects presented mean number of lapses of 1.69 ± 0.24 and the most synchronised ones of 1.53 ± 0.20 without significant differences (*P* = 0.61).

#### Interaction between shift day and IS and between the shift day and BMI

Table 2 shows the effect of interactions between the shift day and IS and between the shift day and BMI on the number of lapses in attention over the seven days evaluation period (Model 1). In the analysis between day of shift work and IS, less synchronised individuals had a greater number of lapses in attention on D7 than D1 (2.60 ± 0.54 and 0.46 ± 0.10, respectively; *P* < 0.001). Also, in the more synchronised group, the highest number of lapses in attention occurred on D7 (2.77 ± 0.43), significantly higher than D1, D2 and D3 (0.67 ± 0.09, 1.66 ± 0.31 and 1.60 ± 0.27, respectively; *P* < 0.001) (Table 2).

**Table 2:** Generalised estimating equation models for effects of shift day, interdaily stability and BMI on lapses in attention of alternating shift workers.

Variable (*n*)	D1	D2	D3	D4	D6	D7
	M (SE)	M (SE)	M (SE)	M (SE)	M (SE)	M (SE)
IS*						
Less synchronised (13)	0.46 (0.10)^a^	2.04 (0.65)^a.b^	2.47 (0.60)^a,c^	1.98 (0.37)b,c	1.94 (0.60)^a,b,c^	2.60 (0.54)^b,c^
More synchronised (12)	0.67 (0.09)^a^	1.66 (0.31)^b^	1.60 (0.27)^b^	1.44 (0.39)^a,b,c^	1.82 (0.42)^a,b,c^	2.77 (0.43)^c^
BMI*						
Eutrophic (5)	0.18 (0.05)^aβ^	0.74 (0.21)^a^	1.24 (0.15)^a^	2.46 (0.33)^b^	1.26 (0.30)^b^	3.00 (0.35)^b^
Overweight (16)	1.81 (0.50)	1.58 (0.51)	1.40 (0.47)	0.92 (0.39)	1.66 (0.53)	2.26 (0.61)
Obese (9)	0.54 (0.00)^β^	5.32 (1.84)	4.55 (1.22)	2.11 (0.47)	3.17 (1.32)	2.85 (0.73)

Note: Data are expressed as mean (standard error, SE). IS, interdaily stability; BMI, body mass index; D, day. *P < 0.01, calculated by generalised estimating equation test. Model 1: dependent variable: lapses of attention; independent variables: day of shift work, BMI and IS. The isolated effects of the independent variables and the interactions between them were tested in the model. Adjusted for age, period of shift work and children at home. The values in a line with different superscripts are significantly different, *P* < 0.05. ^a, b, c^ and ^d^, intragroup differences; ^β^, diferences between groups.

Throughout the work schedule, eutrophic individuals had higher values of lapses in attention on D7 (3.00 ± 0.35) than D1, D2 and D3 (0.18 ± 0.05, 0.74 ± 0.21 and 1.24 ± 0.15, respectively; *P* < 0.001) (Table 2). We did not find differences in the overweight group in the lapses in attention values throughout the work schedule (*P* > 0.05). Also, the number of lapses in attention of the obese individuals was higher on D1 than the eutrophic ones (0.54 ± 0.00 and 0.18 ± 0.05, respectively; *P* < 0.001).

Obese individuals with low synchronisation had a mean number of lapses in attention of 3.41 ± 1.20, while obese ones that were more synchronised presented a mean of 1.85 ± 0.42 (*P* = 0.86). In the less synchronised eutrophic group, the mean was 0.79 ± 0.10 and for the more synchronised group the value was 1.45 ± 0.00 (*P* = 0.17). Less synchronised overweight individuals presented a mean of 1.79 ± 0.39 and more synchronised a mean of 1.34 ± 0.44 (*P* = 0.92).

The analysis of Model 2 found a significant effect of the variables day of the shift rotation, BMI and IV on number of lapses in attention (*P* < 0.001). In relation to the day of the shift schedule, the volunteers had a higher value of lapses in attention on D7 – the last day of the schedule and the night shift (2.19 ± 0.42) – than the first day of the schedule (0.60 ± 0.10, *P* < 0.001). D1 was also lower than D2 and D3 (1.71 ± 0.29 and 2.08 ± 0.34, respectively; *P* < 0.05). No significant isolated effect of activity-rest fragmentation (IV) on the number of lapses in attention was found; less fragmented individuals presented mean lapses of 1.33 ± 0.17 and the more fragmented of 1.38 ± 0.19 (*P* = 0.86).

#### Interaction between shift day and IV and between the shift day and BMI

The analysis of the interaction between day and BMI showed that the eutrophic group presented higher values of lapses in attention on D3 (1.01 ± 0.16) compared to days D1, D4 and D6 (0.18 ± 0.54, 0.5 ± 0.04 and 0.35 ± 0.05, respectively; *P* < 0.001) (Table 3). The number of lapses in attention in the overweight group did not present a significant difference between the days analysed. Obese subjects presented higher values of lapses in attention on D3 (5.64 ± 1.41), with a significant difference in comparison with D1 (0.45 ± 0.08; *P* < 0.001) (Table 3).

**Table 3:** Generalised estimating equation models for effects of shift day, intradaily variability and BMI on lapses in attention of alternating shift workers.

Variable (*n*)	D1	D2	D3	D4	D6	D7
	M (SE)	M (SE)	M (SE)	M (SE)	M (SE)	M (SE)
IV*						
Less fragmented (13)	1.10 (0.29)	0.92 (0.25)	1.43 (0.28)	1.76 (0.34)	1.70 (0.58)	1.27 (0.37)
More fragmented (12)	0.33 (0.66)^a,b^	3.17 (0.63)^a,b^	3.03 (0.80)^a,b^	0.80 (0.19)^a,b^	0.83 (0.17)^a^	4.94 (1.33)^b^
BMI*						
Eutrophic (5)	0.18 (0.54)^a^	0.74 (0.21)^a,b,c^	1.01 (0.16)^b^	0.50 (0.04)^c^	0.35 (0.05)^a,c^	3.00 (1.24)^a,b,c^
Overweight (16)	2.73 (0.95)	1.41 (0.39)	1.59 (0.63)	0.87 (0.32)	1.59 (0.56)	2.42 (0.63)
Obese (9)	0.45 (0.08)^a^	4.83 (1.56)^a^	5.64 (1.41)^b^	3.88 (1.02)^a^	2.94 (1.37)^a^	1.70 (0.59)^a^

Note: Data are expressed as mean (standard error, SE). IV, intradaily variability; BMI, body mass index; D, day. *P < 0.01, calculated by generalised estimating equation test. Model 2: dependent variable: lapses of attention; independent variables: day of shift work, BMI and IV. The isolated effects of the independent variables and the interactions between them were tested in the model. Adjusted for age, period of shift work and children at home. The values in a line with different superscripts are significantly different, *P* < 0.05. ^a, b, c^ and ^d^, intragroup diferences.

The results of Model 2 for the interaction between day and IV showed, for the more fragmented group, that D7 was significantly higher than D6 (4.94 ± 1.33 and 0.83 ± 0.17, respectively, *P* < 0.001) (Table 3). In less fragmented individuals, no significant differences were found (*P* > 0.05) (Table 3).

We also found in Model 2 that the group of more fragmented obese individuals presented a mean number of lapses in attention of 3.83 ± 1.29 and among less fragmented individuals the mean was 1.61 ± 0.34 (*P* = 0.51). For the less fragmented eutrophic group, we found a mean of 1.09 ± 0.25 and in more fragmented individuals the value was 0.25 ± 0.00 (*P* = 0.11). In less fragmented overweight individuals mean lapses in attention were 1.33 ± 0.30 and for more fragmented overweight it was 2.05 ± 0.77 (*P* = 0.70).

## Discussion

This study evaluated the effect of rotating shift work, circadian misalignment and nutritional status on psychomotor performance throughout a complete shift schedule under real life conditions. We found that the shift, obesity, synchronisation and fragmentation of the rest and activity pattern had a significant effect on lapses of attention throughout the scheduled shift days, which supports the hypothesis of the present study. In addition, we found a moderate correlation between lapses in attention in the total shift schedule and BMI. Overall, eutrophic subjects – both less and more synchronised, as well as less or more fragmented activity rhythm – presented a lower number of lapses in attention on several days of the rotating shift schedule when compared with obese/overweight ones. Taken together, the above results demonstrate that psychomotor performance may be influenced by nutritional, work and circadian aspects of the shift worker, which demonstrates the need for a multifactorial approach in interventions aimed at improving the performance and safety of these individuals.

In our study, the number of lapses of attention did not differ between the beginning and end of each day of the schedule, but rather with the advancement of the rotation, and especially when the work was performed at night. In this regard, the second day of night shift and the last day of the shift schedule (D7) presented the most impairment in terms of psychomotor performance compared to other days (Tables 2 and 3). The negative effects of night work on performance have already been recognised in previous studies^36–39^. Magee *et al*.^36^ showed a greater deleterious effect on alertness and neurobehavioural performance when individuals worked consecutive night shifts. Folkard and Lombardi^37^ found that during the second day of night shift the risk of work accidents increased by 6% compared to the first day of the night shift. A study conducted by Keith *et al*.^38^ found a decrease in workers’ cognitive performance during the night shift when compared with the morning shift. Reinke *et al*.^39^ also observed that the occurrence of lapses in attention increased during night shifts. Our results corroborate previous studies, which reaffirmed the deleterious effect of night shift on performance. Given that the number of lapses in attention can be influenced by “time of day” and be impacted by sleepiness and fatigue, it is important to consider the impact of these results on human errors and, consequently, the high levels of occupational accidents^40^. Therefore, intervention strategies should be adopted in individuals working at night to prevent deleterious effects on psychomotor performance and ensuring the safety of these workers.

The results from this study showed that the second day of night work (D7) represented the worst day in terms of psychomotor performance for both groups. It is also worth noting that the values of lapses of attention oscillated more along the rotation of shifts in the more synchronized group (>IS) than in the less synchronized group (<IS) (see results-Table 2). Less synchronization - in other words, low values of IS - is a repercussion of a possible misalignment between photic and non-photic synchronisers (eg shift work) with circadian functions (eg sleep-wake cycle)^33^, and this mechanism may explain the largest fluctuations in the number of lapses in the more synchronized group. This is because individuals with higher IS have their activity-rest rhythm more synchronized to the light-dark cycle, which results in better established sleeping and waking times^9^. Thus, these individuals may have more difficult adapting to quick time alerting on a rotating shift schedule^9,41,42^. In addition, it should be noted that we use the median IS values to determine the group division and we haven’t determined if this value is the most appropriate to classify individuals as more or less synchronized. So far, it has not been described what would be an ideal cutoff point for this analysis, and further studies on this subject are needed.

The rhythmicity of some physiological functions may impact cognitive perfomance, such as some hormones functions, which are related to mediating information from the central nervous system, including the SCN, to the peripheral tissues. Many of these hormones have a daytime secretion pattern, thus in misalignment situations the transmission of these biochemical signals compromises the mechanism called clock-hormone-interaction, so different circadian oscillators, present in tissues, can impact cognitive performance^19,21,43^. A similar mechanism occurs in more fragmented individuals – with higher values of IV – in this case, the fragmentation of the wake-sleep cycle negatively compromises sleep efficiency^33^. Since this cycle is also influenced by the circadian timing system, the misalignment caused by the evening ‘wake-maintenance zone’ (WMZ) and ‘sleep-promoting zone’ (SPZ) leads to an abrupt deterioration of cognitive performance and both situations compromise cognitive performance^12,44^. We predicted that the rotating worker would generally present a lower IS value, but we observed that some volunteers presented a more regular level of synchronization than others, and this may result in better parameters in psychomotor performance. We predicted that the rotating worker would generally present a lower IS value, but we observed that some volunteers presented a more regular level of synchronization than others, and this may result in better parameters in psychomotor performance. We emphasize that new studies are necessary to confirm these findings.

The results from the present study corroborate the study of Chellapa *et al*.^45^, who performed a protocol of circadian misalignment/alignment in night shift workers. The authors showed the effect of the “circadian misalignment condition” on the decrease in cognitive performance when comparing the first day of evaluations (T1) with the others (T2, T3 and T4)^45^. This data can be considered relevant because, although night work leads to circadian desynchronisation, as explained previously, the effect of night work on cognitive performance seems to occur independently of circadian misalignment. Nonetheless, it is important to recognise the potential interaction between night shift work and circadian effects on psychomotor performance, since all these changes related to misalignment drastically impact all physiological functions that have circadian rhythmicity^40^.

Our results also showed that obese individuals presented higher values of lapses in attention throughout the shift schedule compared with eutrophic ones (Figs. 2, 3A,B). Cook *et al*.^23^ also found that obese individuals had a decrease in psychomotor performance when compared to non-obese. Tsai, Huang and Tsai^46^ presented evidence that the group of obese volunteers presented a slower reaction time (PVT) compared with the control group, which impacts the decrease in cognitive performance of obese individuals. Although we believe that eating habits can affect cognitive performance^17^, other studies^23,47^ suggested that the possible mechanisms that lead to a reduction in cognitive performance in obese individuals are linked to increased oxidative stress, metabolic dysfunctions and systemic inflammation that are capable of interfering with brain functions linked to cognitive performance^23^. In this sense, there is increasing evidence linking neuroinflammation with the pathogenesis of obesity and, consequently, with cognitive decline^47^. Another possible explanation for the relationship between obesity and impaired cognitive performance found in the present study may be the increased risk in sleep-disordered breathing^48^ and poorer sleep quality^49^ in obese individuals. Considering the role of sleep on the psychomotor performance already documented in the literature^48^ we might assume that such sleep disorders - and not directly obesity - could mediate impairments in cognitive performance. In the present study, having sleep disorders was considered an exclusion criterion, but this identification may have failed because volunteers did not perform the polysomnography. Further studies should assess whether the effect of BMI on the number of lapses can be mediated by the sleep pattern.

In the present study we did not find significant effects of interaction between IS/IV and BMI on psychomotor performance (Table 4). There is little evidence that components of cognitive function such as vigilance and the number of lapses of attention may also be impacted by the association between circadian misalignment^50^ and obesity^23^. As recent evidence shows that shift work^34^ is related to negative effects on psychomotor performance^51^, we postulated that these variables – shift work, obesity and circadian misalignment - could interact to produce a worse psychomotor performance, but perhaps the isolated impact of other factors common to shift work- such as sleep deprivation itself- may have hampered this identification. Future research that should be done to elucidate this issue.

**Table 4:** Summarizes the post hoc results of model 1 and 2.

Effect tested on lapses of attention	P value	Differences
**Model 1**		
BMI	*P* < 0.001	*Obese* > *Eutrophic*
DAY	*P* < 0.001	*D7*>*D1, D2, D3 and D4*
IS	*P* = 0.61	—
BMI*DAY	*P* < 0.001	*Eutrophic:* D7 > D1, D2 and D3. *Overweight and obese:* No significant differences. *D1:* obese > eutrophic.
IS*DAY	*P* < 0.001	*Less synchronized*: D7 > D1; *More synchronized*: D7 > than D1, D2 and D3.
BMI*IS	*P* < 0.001	p.s: we did not find significant effects on Post hoc results.
BMI*IS*DAY	*P* < 0.001	p.s: we did not find significant effects on Post hoc results.
**Model 2**		
BMI	*P* < 0.001	Obese/Overweight > Eutrophic
DAY	*P* < 0.01	D7 > D1 D1 < D2 and D3
IS	*P* = 0.70	—
BMI*DAY	*P* < 0.001	*Eutrophic:* D3 > D1, D4 and D6 *Overweight* No significant differences. *Obese:* D3 > D1, D2, D4, D6 and D7
IV*DAY	*P* < 0.001	*More fragmented:* D7 > D6 *Less fragmented* No significant differences.
BMI*IV	*P* < 0.001	p.s: we did not find significant effects on Post hoc results.
BMI*IV*DAY	*P* < 0.001	p.s: we did not find significant effects on Post hoc results.

The regression test using linear values of each variable (IS, IV, BMI), showed an association between IV and lapses (B = 5.70, R^2^ = 0.14, p = 0.004) and BMI and lapses (B = 0.25, R^2^ = 0.14, p = 0.004). However, we did not find significant results for IS and lapses (B = −7.01, R^2^ = 0.14, p = 0.12) throughout the complete rotation of shifts.

This study has limitations. The study was conducted within a sample composed of only male workers; a study including women and with a more relevant sample size is necessary for better understanding of the association between alternating shift work and work performance in the general population. Although the data collected were objective, they were dependent on the collaboration of the participants, since the correct use of the devices and the performance of the tests at all times can determine the quality of the data. Furthermore, the performance tasks were applied in a quiet and empty room to minimise the influence of psychological and behavioural determinants.

## Conclusion

The psychomotor performance of workers was affected by shift rotation, especially in the group of workers that were less desynchronised, with fragmented rhythm and overweight and/or obese. Also, eutrophic individuals – both synchronised and with low fragmentation – performed better than overweight/obese ones; the eutrophic group was impacted only by the night shift, increasing the lapses in attention on those days. Additional studies should be performed to confirm these findings.
